# Impact of Caregiving on Various Aspects of the Lives of Caregivers

**DOI:** 10.7759/cureus.1213

**Published:** 2017-05-02

**Authors:** Babar Irfan, Omar Irfan, Ahmed Ansari, Waris Qidwai, Kashmira Nanji

**Affiliations:** 1 Medicine, Jinnah Sindh Medical University (SMC); 2 Medicine, The Aga Khan University; 3 Medical College, The Aga Khan University; 4 Family Medicine, The Aga Khan University

**Keywords:** caregiving, caregiver, impact, health, pakistan

## Abstract

**Objective:**

This study was designed to assess the impact of caregiving on the lives of the caregivers.

**Methods:**

A cross-sectional study was conducted between July and September 2015 at a teaching hospital in Karachi, Pakistan. Participants who were more than 18 years old and were involved in caregiving (former or current) of a family member were invited to participate in the study. The participants were recruited through consecutive sampling technique. A total of 400 caregivers were interviewed. Written informed consent was obtained from all the participants. A pretest structured questionnaire was used for data collection and included sections on demographic details and impact of caregiving on various aspects of the lives of caregivers. The data was analyzed using SPSS version 19 (IBM, NY, USA).

**Results:**

Information about a total of 400 caregivers (215 men and 185 women) was included in the final analysis. The majority (57.0%) of the participants were aged between 18 and 30 years. About three-fifths (60%) of the participants were single and the majority of the participants were students. Approximately 64% of the participants were currently involved in caregiving and about 48% of the participants responded that caregiving has an overall negative impact on various aspects, such as physical (40.8%), psychological (47.8%), and professional aspects (51.8%) of their lives.

**Conclusion:**

Negative impact of caregiving was observed among caregivers due to extensive demands of caregiving and limited resources. Therefore, it is imperative for health care providers to explore, identify and support caregivers to cope in a better way to the challenging task of caregiving.

## Introduction

A caregiver is someone who provides care to a person who is incapacitated or handicapped [1]. In general, care can be given to anyone; it can be a spouse who has suffered from a myocardial infarction; a parent with Alzheimer's disease; a mother-in-law with a malignancy, or a child with traumatic injury caused during sports. Informal caregivers include family members and friends who provide care to their dear ones without any financial benefits; whereas, formal caregivers are volunteers or paid care providers associated with a service system [1].

Women constitute more than half (59% to 75% )of the caregiver population [2]. Evidence from studies suggests that women caregivers are usually involved in physically demanding tasks (i.e. bathing, toileting, and dressing) as compared to their male counterparts, who are more likely to provide financial support [3].

During the period of caregiving, the caregivers experience stress and burden resulting from the rigorous activity of caregiving, which can have a negative impact on their physical, psychological, and social lives, thereby decreasing their quality of life (QOL) [4]. Thus, caregivers are at risk of developing psychiatric disorders [5]. Caregiver burden has been proven to account for poor physical and emotional health [5]. Studies on caregivers suggest that caregiving is associated with psychological complaints such as depression and poor physical and psychological QOL [6-8]. A recent study concluded that caregiving was associated with distress, anxiety, stress, and depression [9].

Caregiving also has positive outcomes such as appreciation from patients, improved family cohesion, developing resilience, and gaining a sense of self-worth and accomplishment [10]. These positive aspects of caregiving have been found to be associated with lower levels of caregiver burden for life [11].

There is limited data regarding impact of caregiving on the lives of the caregivers in Pakistan. Therefore, this study was designed to identify the impact of caregiving on various aspects of the lives of the caregivers.

## Materials and methods

A cross-sectional study was conducted between July and September 2015 at a teaching hospital in Karachi, Pakistan. A total of 400 participants who were 18 years or older and were currently or formerly involved in caregiving to a family member were recruited through consecutive sampling technique. Written informed consent was obtained from the study participants after explaining the study protocol to them. The study protocol was reviewed and approved by the Departmental Research Committee of the Aga Khan University.

A pretested structured questionnaire was used as the data collection tool. The questionnaire was formed after thorough literature search and was reviewed by the authors. It was administered in both English and Urdu languages. The questionnaire was composed of two sections. The first section included demographic details of the participants such as age, gender, educational status, occupation, current caregiver, time since caregiving, and relation with the care recipient. Section 2 of the questionnaire included impact of caregiving on different aspects (physical, psychological, family, professional, and social) of the lives of the caregivers. The data was entered and analyzed using SPSS version 19.0 (IBM, NY, USA). Frequencies were obtained for all variables of interest. The results were presented in the form of frequencies and percentages.

## Results

Information from a total of 400 participants was included in the final analysis. Table 1 presents the sociodemographic characteristics of the study participants. About 54% of the participants were male and 46% were female. Amongst the participants, the majority (57%) were between 18 and 30 years of age. Slightly under 59% of the participants were single. Sixty-four percent of the participants responded that they were currently involved in caregiving while 36% of the participants responded that they were involved in caregiving in the past five years. Most of the caregivers informed that they were giving care to their parents or grandparents; 39% were giving care to their mother and 26% were giving care to their father.

**Table 1:** Sociodemographic Characteristics of Study Participants (n=400)

Variables	Frequency	Percentage
Age		
18-30	228	57.0
31-40	44	11.0
41-50	69	17.0
51-60	47	12.0
61-70	12	3.0
Gender		
Male	215	53.8
Female	185	46.2
Marital status		
Single	236	59.0
Married	164	41.0
Care status		
Current	256	64.0
In the past five years	144	36.0
Duration of Care		
Less than six months	122	30.5
Six months to three years	69	17.3
More than three years	209	52.2
Occupational Status		
Employed	183	45.7
Unemployed	9	2.3
housewife/student	208	52.0
Educational Status		
Up to primary	19	4.8
Matric & intermediate	180	45.0
Graduate	116	29.0
Post Graduate	85	21.2

Table 2 depicts the impact of caregiving on the life of caregivers. About 46% of the participants responded that caregiving had adversely impacted their life. The daily routine, such as sleep, eating, exercise, of the majority of the participants (65%) were affected by caregiving. In addition, caregiving had an adverse impact on the physical (40.8%), psychological (47.8%), family (48.5%), work (51.3%) and social (53%) life of the participants.

**Table 2:** Responses of the Participants on the Impact of Caregiving on the Life of Caregivers (N=400)

*Questions/Response*	*n*	*%*
*Caregiving adversely impact life of caregiver?*		
Yes	184	46.0
No	166	41.5
Not sure	50	12.5
*Caregiving adversely impact physical life of caregiver?*		
Yes	163	40.8
No	191	47.8
Not sure	46	11.4
*Caregiving adversely impact psychological life of caregiver?*		
Yes	191	47.8
No	154	38.5
Not sure	55	13.7
*Caregiving adversely impact family life of caregiver?*		
Yes	194	48.5
No	152	38.0
Not sure	54	13.5
*Caregiving impacts professional/work life of caregiver?*		
Yes	205	51.2
No	143	35.8
Not sure	52	13.0
*Caregiving adversely impact social life of caregiver?*		
Yes	212	53.0
No	144	36.0
Not sure	44	11.0
*Caregiving adversely impact financial status of caregiver?*		
Yes	194	48.5
No	149	37.3
Not sure	57	14.2
*Caregiving adversely impact daily routine of caregiver such as sleep, eating, exercise?*		
Yes	262	65.5
No	98	24.5
Not sure	40	10.0
*Caregiving adversely impact recreational life of caregiver?*		
Yes	206	51.5
No	137	34.3
Not sure	57	14.2
*Caregiver often neglects his/her own health during caregiving?*		
Yes	245	61.3
No	102	25.5
Not sure	53	13.2

## Discussion

Caregiving includes assistance with activities of daily living, helping in medical care (e.g. medication management and accompanying patients to the hospital), and providing emotional and financial support [12]. In our study, the majority of the caregivers were males, that is, they were mainly providing the financial support. This is consistent with our tradition and culture where males provide financial support to the family as mostly they are the sole bread earners. Moreover, the majority of the caregivers were students enrolled in universities for graduation or post-graduation degrees. The potential negative effects on caregivers can extend well beyond mental and physical health. A study demonstrated that caregivers, particularly younger caregivers, often neglect their education, putting education on hold or dropping out entirely, which can impact their future career [13].

Almost half of the participants in the study responded that they suffered from financial difficulties while providing care; similar responses were recorded from a survey which concluded that caregiving results in considerable financial strain, and can cause difficulties in fulfilling other responsibilities such as marriage and employment [14]. In the current study, about 51.3% individuals reported that their professional work life has been negatively affected due to caregiving. It is believed that a good income and a high socioeconomic status can have a decreased impact on the caregivers' financial constraints.

Studies have shown that illness of a close relative causes distress and compromises the caregiver’s health to a greater extent [15]. We report that 61.3% individuals in the study neglected their health during caregiving. This is consistent with the results of previous studies which had concluded that caregivers may be particularly vulnerable because of caregiving demands that can lead to compromising their physical health and can exacerbate existing chronic health conditions [16]. Dementia caregivers report more depression than any other caregivers [17]. Due to lack of time, the caregivers are less likely to care for themselves and may be prone to certain medical conditions [18].

Our study results show that caregiving has an overall impact on daily life as 65% of the participants reported to have altered sleeping and eating habits. A meta-analysis of the physical and mental health effects of caregiving have concluded that higher levels of depression and physical health problems were observed among caregivers as compared with non-caregivers [19].

Some studies have shown contrasting results emphasizng the positive effects of caregiving. These studies report that caregivers may even experience improved health status because of their caregiving experience. However, there are clearly subgroups of caregivers who report high levels of stress and experience depression from caregiving.

Caregiving can be a very challenging task where caregivers do not have enough resources such as knowledge, skills, social support and face a lack of respite and community services.

The study had several potential limitations. Since this was a cross-sectional study, causality was difficult to establish. Moreover, in this study we did not focus on a specific disease or ailment, as the caregiver burden and impact might get changed due to different diseases. The data was collected from an urban city of Pakistan; therefore, the results may vary when compared to the data from people living in rural areas.

## Conclusions

In conclusion, our study shows the negative impact of caregiving on various aspects of the lives of the caregiver. This could provide an evidence basis for the development of support programs designed for caregivers. Health care professionals should be prepared to address the caregivers' psychological, social, and health needs. They should also highlight the positive aspects of caregiving such as a feeling of pride, a sense of purpose, and satisfaction received from giving care to dear ones.
